# Differentiating and Categorizing of Liposarcoma and Synovial Sarcoma Neoplasms by Fluorescence in Situ Hybridization

**Published:** 2017-07-01

**Authors:** Farhad Shahi, Razieh Alishahi, Hossein Pashaiefar, Isa Jahanzad, Naser Kamalian, Ardeshir Ghavamzadeh, Marjan Yaghmaie

**Affiliations:** 1 *Dept. of Hematology and Medical Oncology, Cancer Research Center, Cancer Institute, Imam Khomeini Hospital Complex, Tehran University of Medical Sciences, Tehran, Iran*; 2 *Dept. of Biology, Faculty of Sciences, International Pardis, University of Guilan, Rasht, Iran*; 3 *Dept. of Medical Genetics, School of Medicine, Tehran University of Medical Sciences, Tehran, Iran*; 4 *Dept. of Pathology, Cancer Research Center, Cancer Institute, Imam Khomeini Hospital Complex, Tehran University of Medical Sciences, Tehran, Iran*; 5 *Dept. of Pathology, Shariati Hospital, Tehran University of Medical Sciences, Tehran, Iran*; 6 *Hematology, Oncology and Stem Cell Transplantation Research center, Tehran University of Medical Sciences, Tehran, Iran*

**Keywords:** Liposarcoma, Synovial Sarcoma, FISH, CHOP, SYT, MDM2

## Abstract

**Background & Objective:**

Soft tissue sarcomas (STS) constitute an uncommon and heterogeneous group of tumors of mesenchymal origin and various cytogenetic abnormalities ranging from distinct genomic rearrangements, such as chromosomal translocations and amplifications, to more intricate rearrangements involving multiple chromosomes. Fluorescence in situ hybridization (FISH) can be used to identify these chromosomal translocations and amplifications, and sub classify STS precisely. The current study aimed at investigating the usefulness of FISH, as a diagnostic ancillary aid, to detect cytogenetic abnormalities such as *MDM2* (murine double minute 2*)* amplification and *CHOP*(C/EBP homologous protein) rearrangement in liposarcoma, as well as *SYT* (synaptotagmin*)* rearrangement in synovial sarcoma.

**Methods:**

The FISH technique was used to analyze 17 specimens of liposarcoma for *MDM2* amplification and *CHOP* rearrangement, and 10 specimens of synovial sarcoma for *SYT* rearrangement. The subtypes of liposarcoma and synovial sarcomas were reclassified according to the FISH results and compared with those of the respective histological findings.

**Results:**

According to the FISH results in 17 liposarcoma cases, well-differentiated liposarcoma(WDLPS), dedifferentiated liposarcoma (DDLPS), and myxoidliposarcoma (MLPS)subtypes were 41%, 53%, and 6%, respectively. In different subtypes of liposarcoma, a total of 30% mismatches were observed between pathologic and cytogenetic results. According to the histological findings from FISH analysis, *SYT* rearrangement was found only in three out of 10 (30%) synovial sarcomas.

**Conclusion:**

The detection of cytogenetic abnormalities in patients with liposarcoma and synovial sarcoma by FISH technique provides an important objective tool to confirm sarcoma diagnosis and sub classification of specific sarcoma subtypes in such patients.

## Introduction

Soft tissue sarcomas (STS) are a biologically complex and remarkably heterogeneous group of uncommon tumors of mesenchymal origin that represent only 1% of all human malignancies (1). These tumors have distinctive histology and a wide spectrum of clinicopathological features. More than 100 different malignant and benign soft tissue neoplasms are classified by the World Health Organization (WHO). This vastness and variety of soft tissue neoplasms makes their diagnosis and classification difficult, and undoubtedly one of the most complex areas in clinical pathology, resulting in a high rate of misdiagnosis and misclassification (2, 3). Although assessment of pathologic subtypes or grades of an individual sarcoma is a means of predicting its clinical behavior and is important to determine therapeutic strategies, it is a frequent diagnostic dilemma; therefore, a disagreement rate of 40% exists between expert pathologists (4). Due to difficulties to diagnose and classify soft tissue sarcomas, molecular methods such as FISH and polymerase chain reaction (PCR)-based techniques are routinely used to diagnose and classify some types of STS as alternative methods(5). The two most important and prevalent soft tissue sarcomas in adults are liposarcomas (LPS) and synovial sarcomas (SS), representing about 17% to 25% and 10% of total cases, respectively (6, 7). 

According to histopathological diagnostic criteria, liposarcomas and synovial sarcomas can be subdivided into four and three main subtypes, respectively; each with its own specific and unique clinicopathological characteristics and behavior. Well-differentiated liposarcoma (WDLPS), dedifferentiated liposarcoma (DDLPS), myxoidliposarcoma (MLPS), and pleomorphic liposarcoma (PLPS) are the main subtypes of liposarcoma; mono phasic, biphasic, and poorly differentiated synovial sarcomas are the main subtypes of synovial sarcomas (8, 9).

The morphological diversity of liposarcomas and synovial sarcomas reflects the variation in their clinicopathological behavior ranging from tumors with low risk for metastasis, such as WDLPS, to tumors with high risk for metastasis, such as the round cell (RC) variant of MLPS or PLPS, and poorly differentiated synovial sarcoma (10, 11). Differential diagnosis is of the critical importance to diagnose and treat liposarcomas and synovial sarcomas. Differentiating liposarcomas from lipomas, synovial sarcomas from fibrosarcomas or leiomyosarcomas, and classification of these types of sarcomas are crucial to provide patients with therapeutic strategies and predict their prognosis, although LPS and SS may not have notable findings on histopathology; the result is a high rate of their misdiagnosis and misclassification.

Several studies showed the potential utility of genetic approaches to detect liposarcoma and synovial sarcoma and their classification (12-15).

Lioposarcomas and synovial sarcomas, similar to many types of soft tissue sarcomas, are associated with specific genetic alterations such as translocations and amplifications, which are helpful to diagnose individual cases (16).

Regarding liposarcomas, *MDM2* gene amplification and *CHOP* gene rearrangement are useful to sub classify liposarcomas, and can be utilized to differentiate certain subtypes of liposarcomas from benign lipomas (17). Primary amplification of *MDM2* is predominantly observed in WDLPS and DDLPS, but not in benign lipomas and PLP cases, making this feature a useful tool to differentiate WDLPS and DDLPS from benign lipomas and PLP (18).* MDM2* amplification is not observed in PLP cases (19).

*CHOP *(*DDIT3*) gene rearrangement is the main feature of myxoidliposarcomas (MLPS) and is observed in nearly all cases of MLPS. A t(12;16), or t(12;22) translocation, leading to fusion of *CHOP *(*DDIT3*) located on 12q13 with *TLS *(*FUS*) on 16p11 or *EWSR1* on 22q12, can be found in nearly all cases of MLPS.

Regarding synovial sarcomas, a t(X;18) translocation is used to directly assist differentiating synovial sarcoma from other STS (20). The translocation fuses *SYT* gene from chromosome 18 to either of the two highly homologous genes at Xp11, SSX1 or SSX2, or in less than 1% of SSX4 cases (21).

These genomic alterations can be detected in patients’ specimens with high accuracy by FISH. Fluorescence in situ hybridization (FISH) is one of the most powerful cytogenetic techniques used by biomedical researchers, and is a routine ancillary tool for pathological diagnosis of different subtypes of STS.

Regarding liposarcomas and synovial sarcomas, FISH is commonly used to detect*MDM2* amplification and *CHOP* rearrangement in liposarcomas and *SYT* rearrangement in synovial sarcomas(18, 22).

The current study used the FISH technique as an ancillary tool to detect *MDM2 *amplification and *CHOP* rearrangement in liposarcomas and *SYT* rearrangement in synovial sarcomas, aiming at differentiating liposarcoma and synovial sarcoma subtypes from other morphologically similar sarcomas and benign conditions. Also, the study investigated the rate of discordance between pathologic and cytogenetic results, and reclassified sarcomas according to cytogenetic results.

## Materials

**Specimens**

A total of 17 liposarcomas and 10 synovial sarcomas archival formalin-fixed, paraffin-embedded (FFPE) tissue blocks were retrieved from the Pathology Department of Cancer Institute, Imam Khomeini Hospital Complex and Kamalian Pathology Lab, from October 2014 to December 2015.

Hematoxylin-Eosin (H&E)-stained slides were prepared, their histopathological features were reviewed by an expert pathologist, and the specimens were classified according to the criteria of the WHO classification system (7).

The specimens consisted of four atypical well-differentiated liposarcomas (WDLS) (14.8%), six myxoidliposarcomas (22.2%), two pleomorphic liposarcomas (7.4%), five unclassified liposarcomas (18.5%), four synovial sarcomas (14.8%), one small round cell synovial sarcoma (3.7%), and five spindle cell tumors in favor of synovial sarcoma (18.5%) (Table1).

**Table 1:** Characteristics of Patients and Tumors

No	Gender	Age (years)	Original Diagnosis	Primary Site	Tumor Size (cm)
1	Male	60	PLPS	Right leg	8
2	Female	50	Liposarcoma	Kidney	10
3	Female	74	MLPS	Abdominal	38
4	Male	57	WDLPS	Abdominal	30
5	Male	63	MLPS	Retroperitoneal	25
6	Male	38	MLPS	Right tight	15
7	Female	48	Liposarcoma	Elbow	1.7
8	Male	74	MLPS	Abdominal	19
9	Male	54	PLPS	Intraabdominal	7.5
10	Male	76	WDLPS	Retroperitoneal	30
11	Male	45	Liposarcoma	Left leg	8
12	Male	20	Liposarcoma	Proximal tibia	5.5
13	Male	72	Liposarcoma	Retroperitoneal	8
14	Male	69	WDLPS	Retroperitoneal	15
15	Female	65	MLPS	Left shoulder	17
16	Male	82	MLPS	Abdominal	7
17	Female	22	WDLPS	Abdominal	40
18	Female	50	Synovial sarcoma	Right chest	13.5
19	Female	28	Small round cell synovial sarcoma	Right forearm	9
20	Female	39	Mono phasic spindle cell sarcoma	Right foot, below knee	17
21	Female	58	Spindle cell tumor in favor of synovial sarcoma	Pelvic	16
22	Male	32	Spindle cell tumor in favor of synovial sarcoma	Left foot	7
23	Male	25	Spindle cell tumor in favor of synovial sarcoma	Right leg	4
24	Female	41	Synovial sarcoma	Right axillary and shoulder	19
25	Female	32	Synovial sarcoma	Abdominal wall	22
26	Male	49	Synovial sarcoma	Chest wall	16
27	Female	34	Spindle cell tumor in favor of synovial sarcoma	Left leg	12

PLPS, Pleomorphic Liposarcoma; MLPS, Myxoidliposarcoma; WDLPS, Well-differentiated Liposarcoma

FISH was performed on inter phase nuclei present on FFPE tissue sections, according to the manufacturer’s instructions. Unstained 3-μm parallel sections were placed on electro-statically positively charged slides (Menzel-Gläster, Braunschweig, Germany). One slide of each patient was stained by H&E and the malignant cell areas were marked by an expert pathologist. The *MDM2 *(12q15) dual-color probe, *CHOP *(12q13) dual-color, break-apart probe, and *SYT* (18q11) dual-color, break-apart probe (Cytocell Aquarius, England) were applied on the marked areas of parallel sections where the malignant cells were present. The hybridized slides were reviewed on an Olympus, BX51 microscope (Olympus, Tokyo, Japan) at x100 magnification with immersion oil using a DAPI/Green/Red triple band pass filter set.

The tissue segments were scored through evaluating a minimum of 100 tumor nuclei per sample. The amplification of *MDM2 *was defined as an *MDM2*/CEP12 ratio of ≥2 in 100 tumor cells. The results were considered positive for *CHOP* and *SYT* when more than 5% of tumor nuclei had evidence of *CHOP* or *SYT* rearrangement.

Regardless of histological classification of samples, they were reclassified according to FISH results and compared with each other. 

## Results

A total of 27 sarcoma tumor specimens, already diagnosed according to histopathological criteria, were analyzed in the current study. They included 17 liposarcomas (63%) and 10 synovial sarcomas (37%). The specimens belonged to 15 males (55.6%) and 12 females (44.4%) with a mean age of 50 years; ranged from 20 to 82.

Table 1 summarizes the tumors histological subtypes, size, and site at the time of diagnosis. The mean and median of tumor size were 16.75 cm and 15 cm (1.7 to 40 cm) in liposarcomas and 13.55 cm and 14.75 cm (4 to 22 cm) in synovial sarcomas cases, respectively. Abdomen and retro peritoneum were the commonest sites of liposarcomas (58.8%), while 70% of synovial sarcomas were located around the limbs. After the initial diagnosis and initiation of treatment, the patients with sarcoma were followed-up. The mean of follow-up period of the patients was 32.3 months (2 to 45 months).

**Figure 1:**
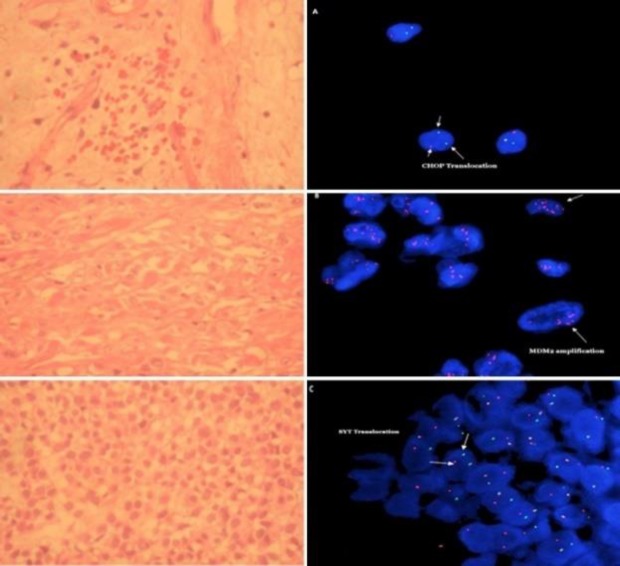
A) CHOP rearrangement in a case with myxoidliposarcoma, ISCN Result: nucish12q13 (CHOPx2) (5′CHOP sep3′CHOPx1) [65/100]

FISH was carried out by commercially available probes for *MDM2* gene amplification and *CHOP* rearrangement in liposarcomas, and for *SYT* rearrangement in synovial sarcomas. The results of FISH were used to reclassify the tumors (Figure 1). 

FISH analysis on a paraffin-embedded tumor with the CHOP probe showed evidence of a 12q13 rearrangement in 65% of inter phase nuclei scored. CHOP rearrangements are recurrent, non-random abnormalities associated with myxoidliposarcomas, and are observed in approximately 95% of cases.

B) MDM2 amplification in a case with liposarcoma

ISCN Result: nucish12q15 (MDM2x3-10), 12cen (D12Z1x2) [60/100]

FISH analysis on a paraffin-embedded tumor with the

MDM2 probe showed evidence of amplification of the MDM2 gene in 60% of interphase nuclei scored.

C) SYT rearrangement in a case with synovial sarcoma

ISCN Result: nucish18q11.2 (SYTx2) (5’SYT sep3’SYTx1) [53/100]

FISH analysis on a paraffin-embedded tumor with the SYT probe showed evidence of an SYT gene rearrangement in 53% of inter phase nuclei scored.

The most common SYT rearrangement is the translocation (X; 18) (p11.2; q11.2), which is a recurrent, non-random abnormality associated with synovial sarcomas and is observed in up to 90% of tumor specimens.

The pathological and FISH results of the patients with liposarcomawere listed in details in Table 2. 

**Table 2:** Revised Diagnosis of Liposarcoma Cases According to FISH Results

*DDIT3* FISH	*MDM2* FISH	Original Diagnosis	Revised Diagnosis
NR	AMP	PLPS	WDLPS
TR	NR	Liposarcoma	MLPS
NR	AMP	MLPS	WDLPS
NR	AMP	WDLPS	WDLPS
NR	NR	MLPS	DLPS
NR	NR	MLPS	MLPS
NR	NR	Liposarcoma	DLPS
NR	AMP	MLP with necrosis	WDLPS
NR	AMP	PLPS	WDLPS
NR	AMP	WDLPS	WDLPS
NR	NR	Liposarcoma	DLPS
NR	NR	Liposarcoma	DLPS
NR	NR	Liposarcoma	DLPS
NR	NR	WDLPS	DLPS
NR	AMP	MLPS	WDLPS
NR	NR	MLPS	DLPS
NR	NR	WDLPS	DLPS

AMP, Amplification; NR, Normal, PLPS, pleomorphic liposarcoma; MLPS, myxoidliposarcoma; WDLPS, well-differentiated liposarcoma; FISH, fluorescence in situhybridization;

*MDM2* amplification was observed in seven cases (41.2%) and *CHOP* rearrangement in two cases (11.8%). According to the results of FISH, the original pathology-based diagnoses were revised in nine cases (52.9%) and all unclassified liposarcomas were successfully classified, including two PLPS reclassified as WDLPS, five Myxoid Liposarcomas(MLS)reclassified as three WDLS and two DDLPS, two WDLPS reclassified as DDLPS, and five unclassified liposarcomas classified as four DDLPS and one MLP.The pathological and FISH results of the patients with synovial sarcoma are shown in Table 3.

**Table 3:** Revised Diagnosis of Synovial Sarcoma Cases According to FISH Results

**Original Diagnosis(OD)**	**Revised Diagnosis(RD)**	***SYT*** ** FISH**
Synovial sarcoma	Normal	NR
Small round cell synovial sarcoma	Synovial sarcoma	TR
Monophasic spindle cell sarcoma	Synovial sarcoma	TR
Spindlecell tumor in favor of synovial sarcoma	Normal	NR
Spindlecell tumor in favor of synovial sarcoma	Normal	NR
Spindlecell tumor in favor of synovial sarcoma	Normal	NR
Synovial sarcoma	Synovial sarcoma	TR
Malignant synovial	Normal	NR
Synovial sarcoma	Normal	NR
Spindle cell tumor in favor of synovial sarcoma	Normal	NR

FISH, fluorescence in situhybridization; NR, Normal

In the cases of synovial sarcomas, *SYT* rearrangement was observed in three cases (30%) 

based on the results of FISH; therefore, the diagnosis of synovial sarcoma was revised in seven cases (70%) and changed to other types of sarcoma.

During the follow-up, the rate of recurrence was82% in liposarcomas and 60% of the patients with synovial sarcoma. 

Regarding liposarcomas, the recurrence rate of WDLPS and DDLPS subtypes were 70% and 85%, respectively.

## Discussion

An accurate diagnosis of different types of STS is important not only to differentiate benign from malignant tumors, but also to predict the behavior of tumors and determine suitable therapeutic strategies.

Although the analysis of histomorphological and immunohistological features is the main procedure for pathological diagnosis of most types of STS, rarity and wide diversity of these malignancies provide specific diagnostic dilemma. Given the tumor-specific genetic alterations elucidated in recent years, molecular analysis has modified the routine diagnostic workup of different types of STS.

Currently, it is estimated that about 30% of sarcomas harbor specific chromosomal abnormalities such as chromosomal translocations and amplifications result in fusion genes; this provides a useful tool for diagnosis and offers novel and potential targets for future therapeutic approaches (23).

Various molecular genetic abnormalities are detected in 12q in different subtypes of liposarcoma including t(12;16)(q13;p11), or t(12;22)(q13;q12) translocations, which lead to fusion of transcription factor gene *CHOP* (*DDIT3*) (a negative regulator of adipocyte differentiation) with *TLS* (*FUS*) or *EWS* genes in at least 95% of MLS cases, as well as amplification of 12q13-15 encompassing *MDM2* and *CDK4* genes in well-differentiated and dedifferentiated liposarcomas(24).

A specific t(X;18)(p11.2;q11.2) translocation resulting in fusion of genes between *SYT*, on chromosome 18, and *SSX1*, *SSX2*, or rarely *SSX4* on chromosome X is detectable in 90% of synovial sarcomas (25, 26). The translocation is found both in the spindle and epithelial components of synovial sarcomas, but not in other spindle cell sarcomas.

FISH is an ideal test to detect such chromosomal abnormalities and differentiate liposarcomas from synovial sarcomas. Specific genetic abnormalities of various sarcoma subtypes, including gene translocation and amplification, can be detected with high sensitivity by FISH.

Recent studies showed that FISH is more specific and sensitive than quantitative polymerase chain reaction (qPCR) and immunohistochemistry (IHC)to detect*MDM2* amplification and *CHOP* rearrangement in liposarcomas and *SYT* rearrangement in synovial sarcomas (27, 28). According to their results, FISH can differentiate different subtypes of sarcomas with high accuracy and sensitivity.

The current study employed the FISH technique to reclassify liposarcomas and synovial sarcomas already classified according to histological features. FISH was performed on 17 liposarcoma and 10 synovial sarcoma tumors with commercially available probes for *MDM2* amplification and *CHOP* and *SYT* rearrangements. FISH results were used to reclassify the sarcoma cases.

According to histopathological findings, the tumors in the liposarcoma group were classified as follows: four WDLPS (23%), six MLPS (35%), and two PLPS (12%), while five tumors (30%) could not be classified according to histopathological features.

Then FISH analysis was performed to determine *MDM2* amplification and *CHOP* rearrangement; therefore, the liposarcoma specimens were reclassified as 41% WLDLP, 53% DDLPS, and 6% MLPS. All the previously unclassified liposarcomas were classified as well with FISH; in details, two PLPS were reclassified as WDLPS, five MLS as three WDLS and two DDLPS, two WDLPS as DDLPS, and five unclassified liposarcomas as four DDLPS and one MLPS.

FISH and histopathological findings were matched in 48.8% for WDLPS, 12% for MLPS, and poorly matched for DDLPS.

According to the histopathological findings, the tumors in the synovial sarcoma group were classified as four synovial sarcomas (40%), one small round cell synovial sarcoma (10%), and five spindle cell tumors in favor of synovial sarcoma (50%);* SYT* rearrangements were observed in only three specimens (30%) with no *SYT* rearrangements in seven specimens (70%). Regarding the synovial sarcoma, there was 20% agreement between FISH and histopathological findings.

These results showed that histopathological findings could not provide conclusive results in 70% of synovial sarcomas and in agreement with previous studies, FISH analysis should be mandatory to accurately diagnose synovial sarcoma and apply appropriate clinical management (29)

Based on the obtained results of FISH performed to detect *MDM2* gene amplification and *CHOP* gene rearrangement in liposarcomas, and *SYT* gene rearrangement in synovial sarcomas, this technique confirmed the diagnosis of such tumors. In particular, detection of such genetic abnormalities with FISH provides means to accurately differentiate the subtypes of liposarcoma from synovial sarcoma.

The current study assessed the specimens already diagnosed as liposarcomas or synovial sarcomas, based on conventional histopathologic examination. After the initial diagnosis and initiation of treatment, the patients with sarcoma were followed-up. The current study results showed that patients with liposarcoma and amplification of *MDM2*had a high rate of recurrence (47%), and patients with *CHOP* rearrangement had no recurrence after treatment. In patients with synovial sarcoma, recurrence occurred after initial operation in two out of three cases with *SYT* rearrangement. In agreement with previous studies, the current study results showed that the detection of these abnormalities by FISH, as an alternative diagnostic approach, is important to predict clinical behavior in patients with liposarcoma and synovial sarcoma (18, 30).

An interesting facet of the current study was that in cases with unclassified liposarcoms without a definitive histological diagnosis, FISH analysis can be used as an ancillary method to accurately diagnose and classify such cases.

In addition to being a diagnostic utility, detection of MDM2 amplification and CHOP *rearrangement* impact liposarcoma treatments that use selective MDM2 inhibitors and blockers of trans-activating ability of FUS-CHOP fusion protein (31). 

In brief, the current study results indicated that FISH analysis of *MDM2* amplification and *CHOP* rearrangement in liposarcomas and *SYT* rearrangement in synovial sarcomas, as well as histopathological findings, were helpful to differentiate such sarcoma subtypes.
